# Novel mechanism of regulation of the 5-lipoxygenase/leukotriene B_4_ pathway by high-density lipoprotein in macrophages

**DOI:** 10.1038/s41598-017-13154-0

**Published:** 2017-10-11

**Authors:** Shigeyasu Tsuda, Masakazu Shinohara, Toshihiko Oshita, Manabu Nagao, Nobuaki Tanaka, Takeshige Mori, Tetsuya Hara, Yasuhiro Irino, Ryuji Toh, Tatsuro Ishida, Ken-ichi Hirata

**Affiliations:** 1 Division of Cardiovascular Medicine, Kobe University Graduate School of Medicine, 7-5-2 Kusunoki-cho, chuo-ku, Kobe, 650-0017 Japan; 2 Division of Epidemiology, Kobe University Graduate School of Medicine, 7-5-2 Kusunoki-cho, chuo-ku, Kobe, 650-0017 Japan; 3 Division of Evidence-based Laboratory Medicine, Kobe University Graduate School of Medicine, 7-5-2 Kusunoki-cho, chuo-ku, Kobe, 650-0017 Japan; 4 The Integrated Center for Mass Spectrometry, Kobe University Graduate School of Medicine, 7-5-2 Kusunoki-cho, chuo-ku, Kobe, 650-0017 Japan

## Abstract

High-density lipoprotein (HDL) interacts with various cells, particularly macrophages, in functional cell-HDL interactions. Here, we found that HDL protein quality and lipid quality play critical roles in HDL functions. HDL fractions from healthy volunteers (HDL_Healthy_) and patients with recurrent coronary atherosclerotic disease (HDL_CAD_) were prepared. To analyse functional HDL-macrophage interactions, macrophages were co-incubated with each HDL, and lipid mediator production was assessed by liquid chromatography/mass spectrometry-based metabololipidomics. HDL_Healthy_ treatment attenuated the pro-inflammatory lipid mediator production, particularly that of leukotriene (LT) B_4_, and this treatment enhanced lipoxin (LX) B_4_ and resolvin (Rv) E2 production. HDL_Healthy_ treatment enhanced the proteasome-mediated degradation of the LTB_4_-producing enzyme 5-lipoxygenase (LO) in activated macrophages; however, HDL_CAD_ did not show these anti-inflammatory effects. HDL_Healthy_ was engulfed by macrophages via clathrin-mediated endocytosis, which was a critical step in 5-LO/LTB_4_ regulation. We also found that HDL_CAD_ showed higher levels of the LTB_4_-producing enzymes and thus promoted LTB_4_ production from HDL_CAD_. In addition, LTB_4_ attenuated HDL endocytosis, HDL-mediated 5-LO degradation in macrophages, and HDL-derived augmentation of macrophage phagocytosis. These results indicated that local LTB_4_ produced *de novo* from HDL_CAD_ regulates HDL-macrophage functional interactions and plays critical roles in dysfunctional, inflammatory HDL characteristics.

## Introduction

High-density lipoprotein (HDL) has diverse anti-atherosclerotic functions, such as reversing cholesterol transport^1^ and inhibiting inflammation^2,3^. Many population studies have shown that the concentration of HDL cholesterol (HDL-C) is inversely related to the risk of coronary atherosclerotic disease (CAD)^4,5^. However, recent unexpected results with inhibitors of cholesteryl ester transfer protein have indicated that pharmacological increases in HDL-C are not necessarily beneficial^6,7^ and that more attention should be focused on HDL function.

The protein quality and lipid quality of HDL play critical roles in HDL function. Recent studies have demonstrated that myeloperoxidase (MPO), a leukocyte-derived haem protein, binds to HDL. MPO generates hypochlorous acid, which oxidizes specific tyrosine and methionine residues on apoA-I and impairs apoA-I-mediated cholesterol efflux^8,9^. In contrast, paraoxonase 1 (PON1), an HDL-associated lipo-lactonase^10^, is linked to the antioxidative, anti-inflammatory, and lipid cargo-carrying functions of HDL^11–13^. We have recently shown that the serum MPO/PON1 ratio may indicate dysfunctional HDL and is useful for risk stratification of CAD patients^14^. Lipid quality, particularly the imbalance between omega-3 and omega-6 fatty acids, may be a risk factor for atherosclerosis^15^. We have reported that eicosapentaenoic acid (EPA)-rich HDL increases cholesterol efflux capacity and PON1 activity, thus indicating that the lipid quality of HDL may regulate its functions^16^.

Chronic inflammation contributes to the development of advanced atherosclerosis^17–19^. The resolution of inflammation is mediated by a phagocytic process by macrophages, known as programmed cell removal or efferocytosis^20^. M2-differentiated macrophages contribute to the resolution of inflammation by producing pro-resolving lipid mediators (LMs) and by producing lower levels of inflammatory LMs, such as LTB_4_ and prostaglandins (PGs), than those produced by M1 macrophages^21^. Although HDL has functional interactions with macrophages during reverse cholesterol transport, little is known about HDL-macrophage interactions with regard to macrophage-dependent LM production. LMs are produced predominantly from polyunsaturated fatty acids, such as arachidonic acid (AA), EPA, and docosahexaenoic acid (DHA), and play crucial roles in the initiation and resolution of inflammatory responses. The balance between pro-inflammatory and pro-resolving mediators regulates the duration of the inflammatory response by promoting neutrophil apoptosis and macrophage efferocytosis^20,22^.

Here, we prepared HDL fractions from healthy volunteers (HDL_Healthy_) and recurrent coronary atherosclerotic disease patients (HDL_CAD_). We found that HDL_Healthy_, through endocytic engulfment into activated macrophages, showed anti-inflammatory effects, thereby limiting pro-inflammatory LTB_4_ production and enhancing anti-inflammatory, pro-resolving LXB_4_ and RvE2 production, as well as enhancing macrophage phagocytosis. Moreover, HDL_CAD_ released *de novo* local LTB_4_, which blocked endocytic engulfment of HDL by macrophages and did not show anti-inflammatory effects. These results provide a novel mechanistic for understanding how HDL_Healthy_ tempers pro-inflammatory responses in HDL-macrophage functional interactions and how HDL_CAD_ becomes dysfunctional or displays pro-inflammatory characteristics.

## Results

### HDL_Healthy_, but not HDL_CAD_, decreases LTB_4_ production from macrophages via proteasome-mediated degradation of 5-LO

To investigate the functional interactions between HDL and macrophages, we prepared HDL from healthy volunteers (N = 4, defined as HDL_Healthy_) and recurrent CAD subjects (N = 4, defined as HDL_CAD_) by ultracentrifugation (Supplemental Data Table 1). Macrophages (RAW 264.7 cell line, 1 × 10^6^ cells) were activated by zymosan (ZyA) (0.1 mg, 30 min at 37 °C), then incubated with HDL_Healthy_ or HDL_CAD_ (10 μg protein, 30 min at 37 °C). We used wide-targeted liquid chromatography (LC)/mass spectrometry (MS)/MS-based metabololipidomics to investigate the LM profiles of HDL-macrophage interactions (Table 1). Macrophages incubated with HDL_Healthy_ produced significantly higher levels of anti-inflammatory/pro-resolving LXB_4_ and RvE2. In contrast, macrophages treated with HDL_CAD_ produced elevated levels of prostanoids, including PGD_2_, PGF_2α_, Thromboxane B_2_, and LTB_4_. As expected from the increases in the levels of PGs and LTs, HDL_CAD_ enhanced phosphorylation of cytosolic phospholipase A2 (cPLA2)-alpha in macrophages (Supplemental Fig. 1). We focused on LTB_4_ because it possesses potent pro-inflammatory activity; however, little is known about its contributions to vascular biology.

**Table 1:** Lipid mediator profiles of macrophages incubated with HDL_Healthy_ or HDL_CAD_.

	Macrophages with HDL_Healthy_ (pg/1 × 10^6^ cells)	Macrophages with HDL_CAD_ (pg/1 × 10^6^ cells)
AA Bioactive Metabolome		
Lipoxin A_4_	0.4 ± 0.2	0.6 ± 0.3
Lipoxin B_4_	5.4 ± 0.4*	—
Prostaglandin D_2_	18434.6 ± 2512.2	35252.7 ± 9233.8*
Prostaglandin E_2_	2185.0 ± 652.6	3205.3 ± 887.5
Prostaglandin F_2α_	3004.3 ± 995.0	7887.6 ± 1096.6*
Thromboxane B_2_	199.0 ± 44.8	1480.6 ± 150.0*
Leukotriene B_4_	1.6 ± 0.3	4.5 ± 2.7*
AA pathway markers		
5 HETE	10.2 ± 3.2	21.6 ± 3.3*
12 HETE	18.9 ± 6.0	11.1 ± 5.9
15 HETE	57.6 ± 16.5	38.1 ± 9.5
EPA Bioactive Metabolome		
Resolvin E1	—	—
Resolvin E2	7.0 ± 3.5*	1.9 ± 0.4
Resolvin E3	—	—
EPA pathway markers		
5 HEPE	5.8 ± 1.8	13.4 ± 5.6
12 HEPE	3.1 ± 1.1	2.5 ± 2.0
15 HEPE	4.0 ± 1.6	4.5 ± 2.9
18 HEPE	5.5 ± 2.0	10.0 ± 4.1
DHA Bioactive Metabolome		
Resolvin D1	1.0 ± 0.3	6.0 ± 1.8*
Resolvin D2	0.6 ± 0.4	—
Resolvin D3	0.5 ± 0.2	—
Resolvin D5	2.3 ± 1.1	0.5 ± 0.4
Maresin 1	—	—
Protectin D1	3.4 ± 1.5	3.0 ± 2.8
DHA pathway markers		
4 HDHA	6.0 ± 2.8	2.9 ± 2.3
7 HDHA	4.0 ± 1.8	1.6 ± 1.2
14 HDHA	2.9 ± 1.2	2.0 ± 1.5
17 HDHA	2.8 ± 0.7	2.1 ± 1.3

— = below detection limits.

Values were represented as mean ± SEM. *P < 0.05 between two groups.

**Figure 1:**
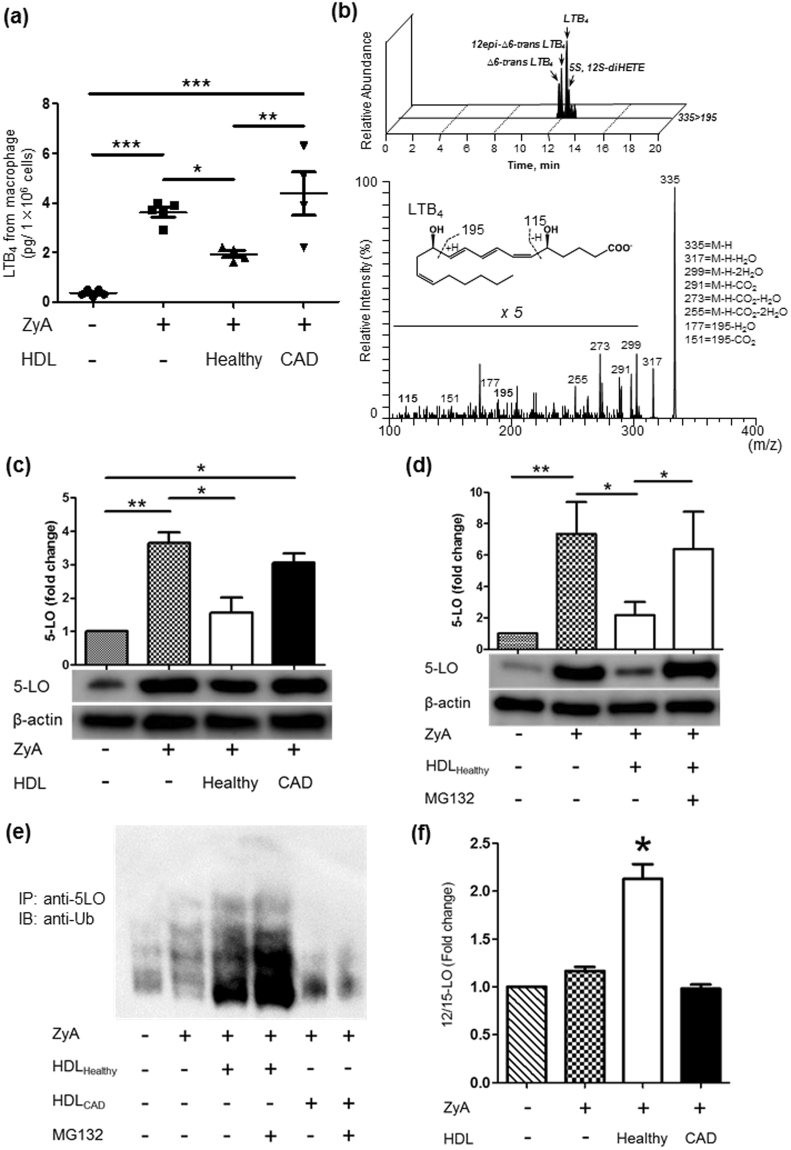
HDL_Healthy_, but not HDL_CAD_, decreased LTB_4_ production from macrophages through proteasome-mediated degradation of 5-LO. Macrophages (RAW 264.7 cell line, 1 × 10^6^) were activated by ZyA (30 min at 37 °C), then treated with HDL_Healthy_ or HDL_CAD_ (10 μg protein). (**a**) LTB_4_ production from macrophages was quantified by LC/MS/MS. The results are expressed as pg/1 × 10^6^ cells, mean ± SEM, N = 4-5 in each group. (**b**) Representative MRM-chromatograph and MS-MS spectrum are presented for the identification of LTB_4_. (**c**) Macrophage lysates were processed for western blot analysis of 5-LO. The results are shown as fold changes from N = 4 experiments. (**d**) 5-LO degradation by HDL_Healthy_ is proteasome-dependent. ZyA-activated macrophages were pretreated for 30 min with the proteasome inhibitor MG 132 (1 µM) or vehicle, then incubated with HDL_Healthy_ for 30 min at 37 °C. Lysates were collected for western blot analysis of 5-LO. The results are shown as the mean ± SEM from N = 4 experiments. *P < 0.05, **P < 0.01, ***P < 0.005. (**e**) HDL_Healthy_ enhanced ubiquitination of 5-LO in macrophages. ZyA-activated macrophages were pretreated for 30 min with the proteasome inhibitor MG 132 (1 µM) or vehicle, then incubated with HDL_Healthy_ or HDL_CAD_ for 30 min at 37 °C. Lysates were immunoprecipitated with anti-5LO antibody (#3289 S, Cell Signaling), using a Dynabeads Protein A IP Kit (Thermo Fisher Scientific), and then immunoblotted with anti-ubiquitin antibody (ab140601, Abcam). (**f**) HDL_Healthy_ enhanced 12/15-LO expression in macrophages. Macrophages (RAW 264.7 cell line, 1 × 10^6^) were activated by ZyA (30 min at 37 °C), then treated with HDL_Healthy_ or HDL_CAD_ (10 μg protein, 30 min at 37 °C). After incubation, total RNA was extracted, and then, 12/15-LO expression was analysed by real-time PCR. The results are shown as fold change compared with the vehicle group; mean ± SEM, N = 4. *P < 0.05 vs the other groups.

Unstimulated macrophages showed limited baseline LTB_4_ production (0.32 ± 0.13 pg/1 × 10^6^ cells, Fig. 1a). After ZyA activation, the levels of LTB_4_ production from macrophages reached 3.54 ± 0.55 pg/1 × 10^6^ cells, and rigorous identification of LTB_4_ was achieved by the LC/MS/MS multiple-reaction monitoring chromatography and MS/MS spectra with more than six signature fragmentations (Fig. 1b). Notably, LTB_4_ as well as its pathway markers (∆6-trans LTB_4_, 12epi-∆6-trans LTB_4_ and 5 S, 12S-diHETE) were also identified, thus indicating distinct activation of the 5-LO-LTB_4_ pathway in macrophages. The interaction between HDL_Healthy_ and macrophages showed significantly lower biosynthesis of LTB_4_; however, HDL_CAD_ did not show lower LTB_4_ biosynthesis (Fig. 1a). LTB_4_ production by mouse bone marrow-derived macrophages was also suppressed significantly by HDL_Healthy_ (Supplemental Fig. 2).

After showing that HDL_Healthy_ regulates LTB_4_ production from macrophages, we next focused on the protein expression of 5-LO, because it is a key enzyme in LTB_4_ biosynthesis from arachidonic acid. Western blot analysis revealed that stimulation of macrophages with ZyA augmented 5-LO protein expression (Fig. 1c, 2nd lane). HDL_Healthy_ showed lower 5-LO protein expression in ZyA-activated macrophages (Fig. 1c, 3rd lane), whereas HDL_CAD_ did not show a significant decrease in 5-LO protein expression (Fig. 1c, 4th lane). We hypothesized that ubiquitin-proteasome degradation was involved in the HDL-mediated decrease in 5-LO. To confirm this possibility, we used the proteasome inhibitor MG-132^23^. Pretreatment of macrophages with MG132 (1 µM, for 30 min) inhibited HDL-mediated 5-LO reduction (Fig. 1d, 4th lane); HDL_Healthy_ also enhanced ubiquitination of 5-LO (Fig. 1e), thus indicating that HDL_Healthy_ decreased 5-LO through ubiquitin-proteasome degradation. Moreover, HDL_Healthy_ enhanced 12/15-LO mRNA expression in macrophages (Fig. 1f), thereby indicating that HDL_Healthy_ induced the enzymatic pathway of pro-resolving mediator biosynthesis. HDL_Healthy_ also suppressed production of M1-related cytokines, namely, IL-6, TNF-α, and IL-1β (Supplemental Fig. 3). However, macrophages showed pro-inflammatory cytokine profiles by HDL_CAD_. Together, these results indicated that HDL_Healthy_, but not HDL_CAD_, decreased LTB_4_ production in macrophages by proteasome-mediated degradation of 5-LO and enhanced specialized resolving mediator release.

### HDL_Healthy_ particle engulfment and localization in macrophages through clathrin-mediated endocytosis

**Figure 2:**
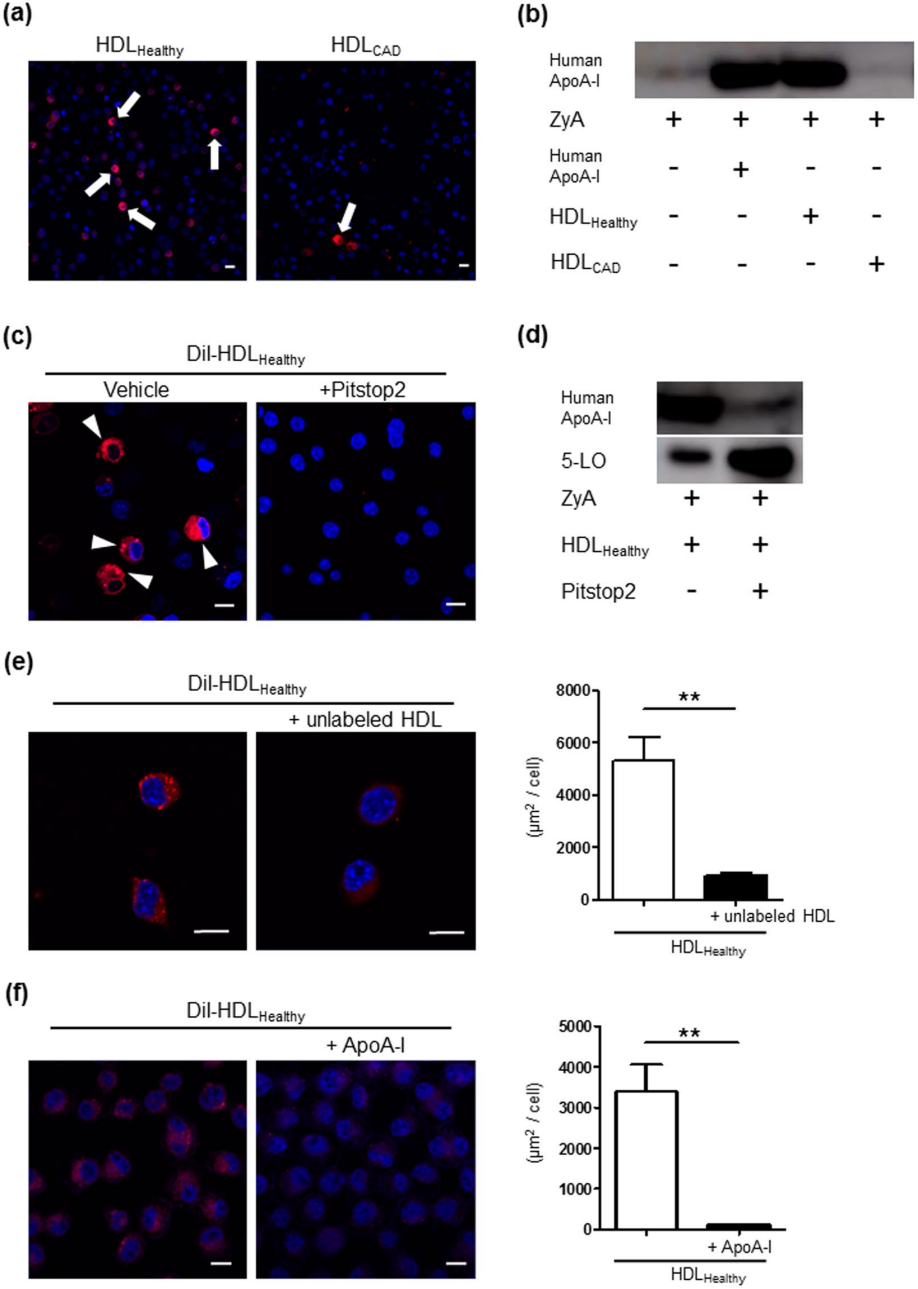
HDL_Healthy_, but not HDL_CAD_, was engulfed by macrophages through clathrin-mediated endocytosis. (**a**) Macrophages were incubated with DiI-labelled HDL (shown as red) for 30 min at 37 °C, then subjected to nuclear staining with DAPI (shown as blue). The arrows indicate HDL engulfment into macrophages (Scale bar = 10 μm). (**b**) Activated macrophages (1 × 10^6^) were co-incubated with human apoA-I (10 µg) or each HDL (10 µg) for 30 min at 37 °C. After extensive washing with PBS^−/−^, macrophages were harvested. Cell lysates were processed for western blot analysis of human apoA-I. The image is representative of results from N = 3 experiments. (**c**) Macrophages were incubated with DiI-labelled HDL (10 µg) with or without 30 min pretreatment with the clathrin-mediated endocytosis inhibitor Pitstop 2 (20 μM). The arrowheads indicate HDL engulfment into macrophages (Scale bar = 10 μm). (**d**) Activated macrophages (1 × 10^6^) were treated with HDL_Healthy_ (10 µg) for 30 min at 37 °C with or without pretreatment with 20 μM Pitstop 2. After extensive washing, cell lysates were processed for western blot analysis of apoA-I and 5-LO. The image is representative of results from N = 3 experiments. (**e**) HDL_Healthy_ engulfment was competitively blocked by unlabelled HDL. Macrophages were incubated with DiI-labelled HDL (shown as red) alone or in the presence of 40-fold excess of unlabelled HDL for 30 min at 37 °C, and this was followed by DAPI staining (shown as blue). The DiI-positive area in the macrophages was analysed by ImageJ as μm^2^/cell, N = 6. (**f**) Macrophages were incubated with DiI-labelled HDL_Healthy_ (shown as red) in the presence or absence of a 40-fold excess of unlabelled apoA-I for 30 min at 37 °C, (Scale bar = 10 μm), and the DiI-positive area in macrophages was analysed by ImageJ as μm^2^/cell. The data are shown as the mean ± SEM of 3 independent experiments. **P < 0.01.

We found that HDL_Healthy_ and HDL_CAD_ have a distinct effect on LM production by macrophages. To investigate the details of HDL-macrophage interactions, we tracked HDL within macrophages, because specific cells other than macrophages have recently been reported to endocytose HDL holoparticles^24–26^. Macrophages were incubated with DiI-labelled HDL for 30 min at 37 °C, and this was followed by nuclear staining with DAPI. Here, we detected the engulfment of HDL_Healthy_ into macrophages; however, low uptake of HDL_CAD_ was observed (Fig. 2a). To confirm the uptake of human-derived HDL into macrophages, we investigated human apoA-I protein expression in the murine macrophage cell line RAW 264.7. After co-incubation with human-derived HDL, macrophages were extensively washed with phosphate-buffered saline, and then the cell lysates were analysed by western blot using an antibody specific to human apoA-I. Negligible human apoA-I protein expression was observed in murine macrophages (Fig. 2b, 1st lane). Co-incubation with human apoA-I protein (10 µg) showed human apoA-I expression in murine macrophages (Fig. 2b, 2nd lane). Macrophages co-incubated with HDL_Healthy_ (10 µg) showed substantial human apoA-I expression (Fig. 2b, 3rd lane); however, co-incubation with HDL_CAD_ (10 µg) did not show detectable human apoA-I protein expression in murine macrophages (Fig. 2b, 4th lane). We also confirmed that the apoA-I protein expression levels were similar between HDL_Healthy_ and HDL_CAD_ (Supplemental Fig. 4).

To address the mechanism underlying macrophage engulfment of HDL_Healthy_, we next used the clathrin-mediated endocytosis inhibitor Pitstop 2^27^. Macrophages were incubated with DiI-labelled HDL with or without pretreatment with 20 μM Pitstop 2 for 30 min. HDL_Healthy_ engulfment into macrophages was confirmed by confocal microscopy (Fig. 2c, left panel), and Pitstop 2 treatment was found to completely abolish HDL uptake (Fig. 2c, right panel). In the same experiment, macrophage cell lysates were obtained and subjected to western blot analysis of apoA-I and 5-LO (Fig. 2d). Pitstop 2 treatment attenuated human apoA-I protein expression in murine macrophages incubated with HDL_Healthy_ (Fig. 2d, upper panel). Additionally, Pitstop 2 treatment augmented 5-LO protein expression within macrophages (Fig. 2d, lower panel). Furthermore, HDL_Healthy_ particle engulfment was competitively blocked by excess unlabelled HDL (Fig. 2e) or apoA-I (Fig. 2f).

These findings indicated that engulfment of HDL_Healthy_ into macrophages is regulated by clathrin-mediated endocytosis, presumably through a specific receptor that binds to apoA-I and that HDL endocytosis regulates HDL-mediated degradation of 5-LO in macrophages. HDL_CAD_ was not endocytosed into macrophages and thus did not regulate 5-LO expression in macrophages. To explore the localization of macrophage-engulfed HDL, we performed immunohistochemical analysis of macrophages with anti-human apoA-I and anti-EEA1 (early endosomal-antigen-1) antibodies (Supplemental Fig. 5a) or anti-human apoA-I and anti-LAMP1 (lysosomal marker) antibodies (Supplemental Fig. 5b). ApoA-I and EEA1 did not co-localize; however, after 120 min of incubation with HDL, we observed partial co-localization of apoA-I and LAMP1 (Supplemental Fig. 5b), thus indicating that the engulfed HDL was localized in lysosomes at this time point.

### *De novo* LTB_4_ production from HDL_CAD_

Local bioactive LMs play critical roles in controlling various macrophage functions^28^. After observing that HDL_Healthy_ and HDL_CAD_ had distinct effects on HDL-macrophage interactions, we examined the differences in the bioactive LMs released from each HDL. Each HDL type (10 µg protein) was incubated with a mixture of 1 µM each of deuterium-labelled AA, EPA, and DHA as substrates for 2 h at 37 °C, and then, deuterium-labelled LM production was analysed by LC/MS/MS. The deuterium-labelling approach enabled us to quantitatively analyse LM production from extra-HDL substrates. The profiles of LM production by HDL_Healthy_ and HDL_CAD_ during the 2-h incubation are shown in Table S2. We found that HDL_CAD_ produced significantly higher levels of PGD_2_ and LTB_4_, as well as 5-LO-related pathway markers, including 5-HETE, 5-HEPE, 4-HDHA, and 7-HDHA, than those produced by HDL_Healthy_.

**Figure 3:**
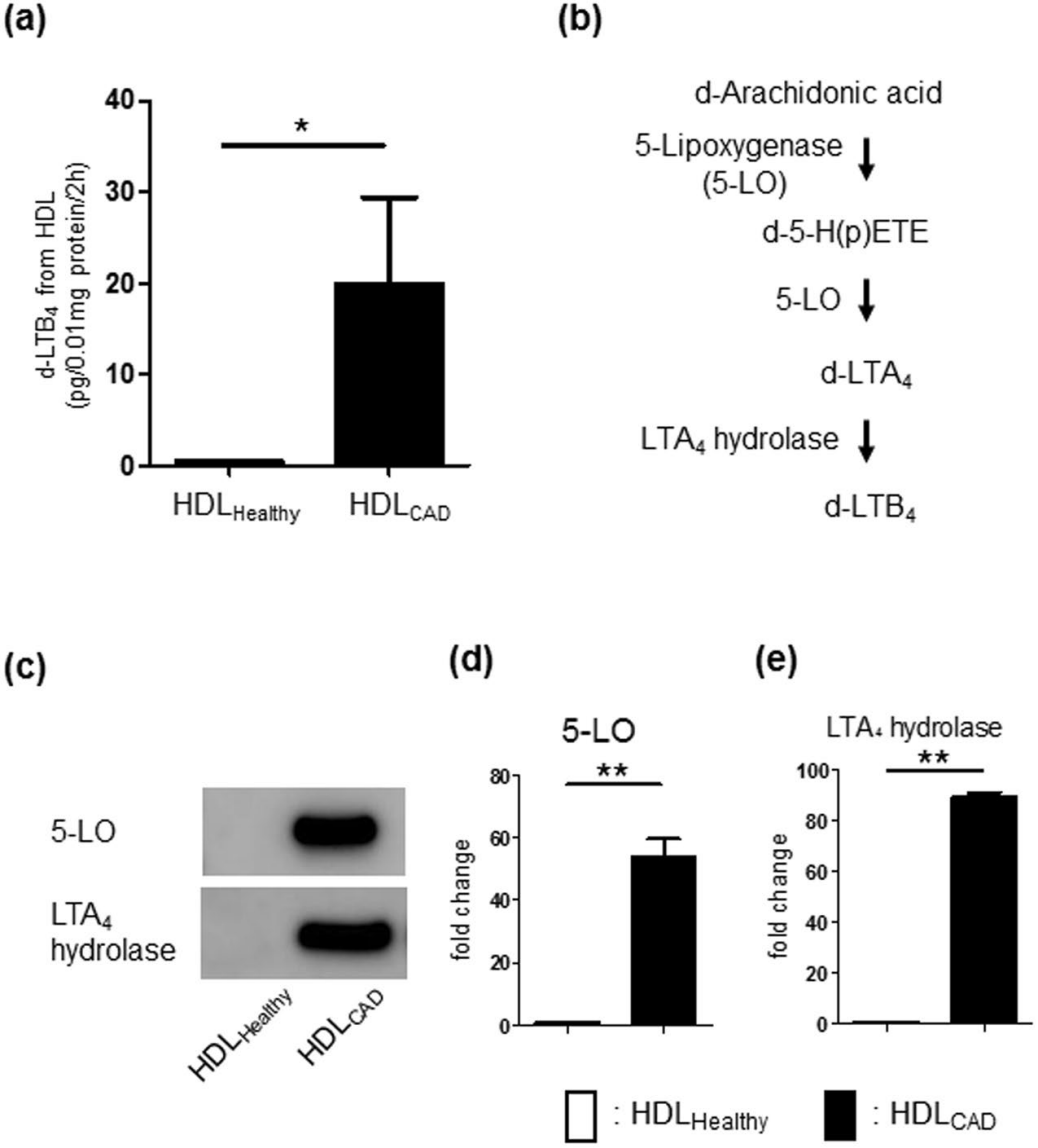
De novo LTB_4_ production from HDL_CAD_. HDL (10 μg) was incubated with 1 µM deuterium-labelled (d-) substrates (d-AA, d-EPA and d-DHA) for 2 h at 37 °C, and then, the biosynthesis of d-LMs was analysed by LC/MS/MS. (**a**) d-LTB_4_ biosynthesis from HDL. The results are shown as pg/0.01 mg of protein/2 h, mean ± SEM, N = 5. *P < 0.05. (**b**) The d-LTB_4_ biosynthesis pathway from d-arachidonic acid is shown. (**c**) Western blot analysis showing 5-LO and LTA_4_ hydrolase in HDL_Healthy_ and HDL_CAD_. Fold changes in the protein expression in HDL_CAD_ compared with those in HDL_Healthy_ are shown. (**d**) 5-LO and (**e**) LTA_4_ hydrolase. The results are shown as the mean ± SEM, N = 3. **P < 0.01.

We focused on *de novo* LTB_4_ synthesis from each HDL (Fig. 3a) and found distinct differences in LTB_4_ production between HDL_Healthy_ and HDL_CAD_. As shown in Fig. 3b, LTB_4_ synthesis from AA requires sequential enzymatic conversion driven by 5-LO and LTA_4_ hydrolase. Next, we investigated whether HDL_CAD_ contains these critical enzymes for LTB_4_ production. Western blot analysis of HDL_Healthy_ and HDL_CAD_ revealed that HDL_CAD_ showed an approximately 55-fold increase in the 5-LO protein levels and an approximately 90-fold increase in LTA_4_ hydrolase protein levels (Fig. 3c–e). Additionally, HDL_CAD_, but not HDL_Healthy_, contained FLAP (Supplemental Fig. 6). These results indicated that HDL_CAD_ carries the neutrophil-like, functional enzymatic machinery that produces pro-inflammatory LTB_4_ from extra-HDL AA.

We hypothesized that exosome components might be transferred to HDL in circulating plasma. To provide evidence for this concept, Plasma_Healthy_ and Plasma_CAD_ were immunoprecipitated with anti-CD9 antibody to collect exosomes using an ExoTrap^TM^ Exosome Isolation Spin Column Kit, and samples were then immunoblotted with anti-CD9 and anti-ApoA-I antibodies. As shown in Supplemental Fig. 7, ExoTrap^TM^ successfully isolated plasma-derived exosomes from each type of plasma (upper panel), and we found that these exosomes also possessed ApoA-I, thus indicating the presence of HDL particles. In this experiment, we did not use separation by ultracentrifugation; therefore, this result suggests that HDL might acquire exosome-derived components during systemic circulation.

### LTB_4_ attenuates HDL_Healthy_ particle engulfment into macrophages and HDL-mediated 5-LO degradation

**Figure 4:**
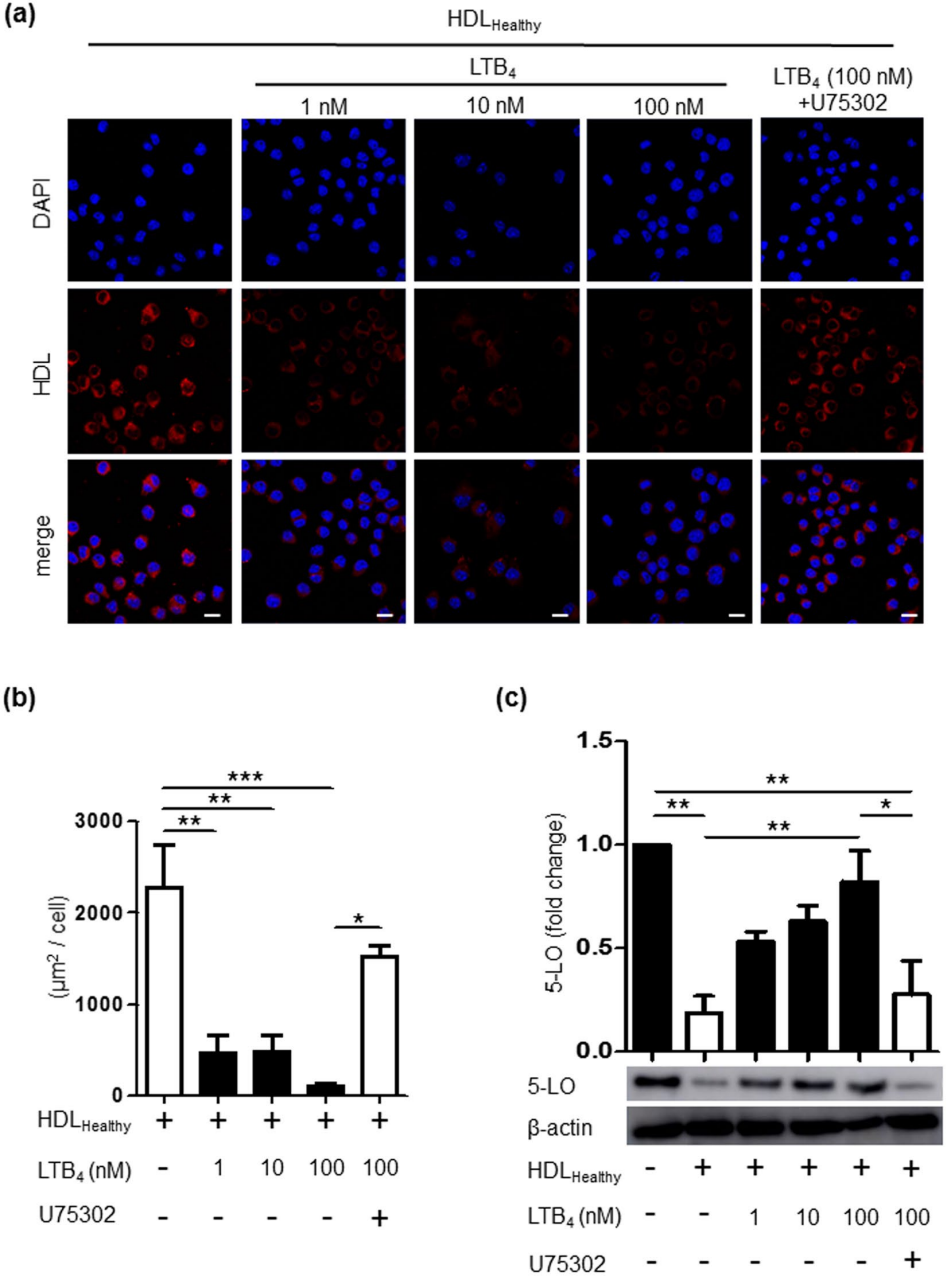
LTB_4_ attenuated HDL engulfment and HDL-mediated 5-LO degradation. (**a**) Macrophages were pretreated for 30 min at 37 °C with the BLT1 inhibitor U75302 (300 nM) or vehicle, and then incubated with LTB_4_ (100 nM) for 30 min at 37 °C and subsequently with DiI-labelled HDL_Healthy_ (shown as red) for 30 min at 37 °C. (**b**) The DiI-positive area in macrophages (μm^2^/cell) was quantified by ImageJ (Scale bar = 10 μm). The data are shown as the mean ± SEM of 3 independent experiments. *P < 0.05, **P < 0.01, ***P < 0.005. (**c**) Macrophages were pretreated for 30 min at 37 °C with U75302 (300 nM) or vehicle and were then incubated with LTB_4_ (1-100 nM) for 30 min at 37 °C and subsequently with 10 μg of HDL_Healthy_ for 30 min at 37 °C. Lysates were collected for western blot analysis of 5-LO. The data are shown as the mean ± SEM of 3 independent experiments. *P < 0.05, **P < 0.01.

Given that HDL_CAD_ locally releases *de novo* LTB_4_, we investigated the effect of HDL-derived LTB_4_ on the functional interactions between HDL and macrophages. Macrophages were incubated with 1-100 nM LTB_4_ for 30 min, then incubated with HDL_Healthy_ for 30 min at 37 °C. Macrophages in specific experiments were pretreated with the LTB_4_ receptor antagonist U75302^29^ (300 nM) for 30 min at 37 °C. We also confirmed BLT1 mRNA expression in RAW macrophages (Supplemental Fig. 8.) Here, we found that 1-100 nM LTB_4_ significantly suppressed HDL engulfment into macrophages (Fig. 4a,b), whereas U75302 treatment rescued HDL engulfment (Fig. 4a,b, 5th lane). LTB_4_-mediated suppression of macrophage engulfment appeared to be specific for clathrin-mediated endocytosis. LTB_4_ selectively attenuated transferrin engulfment into macrophages (Supplemental Fig. 9, upper panels), which was endocytosed via clathrin-mediated machinery^30,31^; however, LTB_4_ had no effect on engulfment of dextran (Supplemental Fig. 9, lower panels), which is internalized via macropinocytosis^32,33^. Notably, LTB4 treatment did not affect the expression of ApoA-I receptors in macrophages, including ABCA1, ABCG1, SR-B1, and SR-A (Supplemental Fig. 10). Next, we investigated whether pretreatment of LTB_4_ might reverse HDL-initiated 5-LO degradation in macrophages. We pretreated macrophages with LTB_4_ (1-100 nM) or vehicle for 30 min, then incubated them with HDL_Healthy_ for 30 min at 37 °C. Cell lysates were processed for western blot analysis of 5-LO (Fig. 4c). We confirmed HDL-mediated reduction of 5-LO (Fig. 4c, 2nd lane). Additionally, 1-100 nM LTB_4_ reversed this HDL-mediated decrease in 5-LO in a dose-dependent manner, resulting in augmented expression of 5-LO in macrophages (Fig. 4c, 3rd-5th lanes). Pretreatment with U75302 (300 nM) inhibited the effects of 100 nM LTB_4_, thus resulting in lower 5-LO expression in macrophages by HDL-mediated degradation (Fig. 4c, 6th lane).

### LTB_4_ receptor antagonist promoted HDL_CAD_ engulfment into macrophages

**Figure 5:**
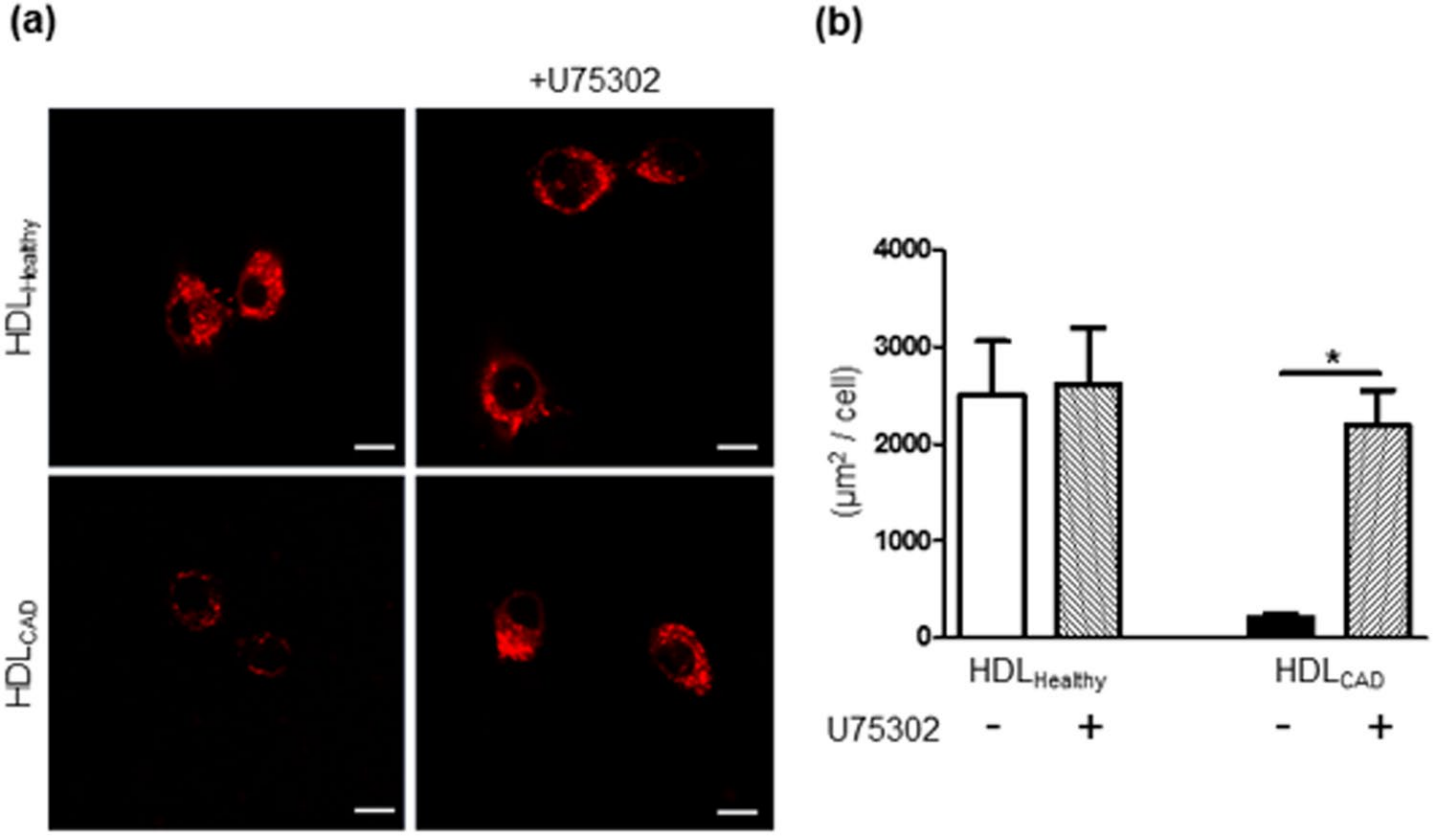
LTB_4_ receptor antagonist promoted HDL_CAD_ engulfment into macrophages. (**a**) Macrophages were pretreated for 30 min at 37 °C with or without LTB_4_ receptor (BLT1) antagonist, U75302 (300 nM) and then were incubated with DiI-labelled HDL_Healthy_ (upper panels) or DiI-labelled HDL_CAD_ (lower panels) for 30 min at 37 °C. Scale bars = 10 μm. (**b**) The DiI-positive area in macrophages (μm^2^/cell) was quantified by ImageJ. The data are shown as the mean ± SEM of 3 independent experiments. *P < 0.05.

**Figure 6:**
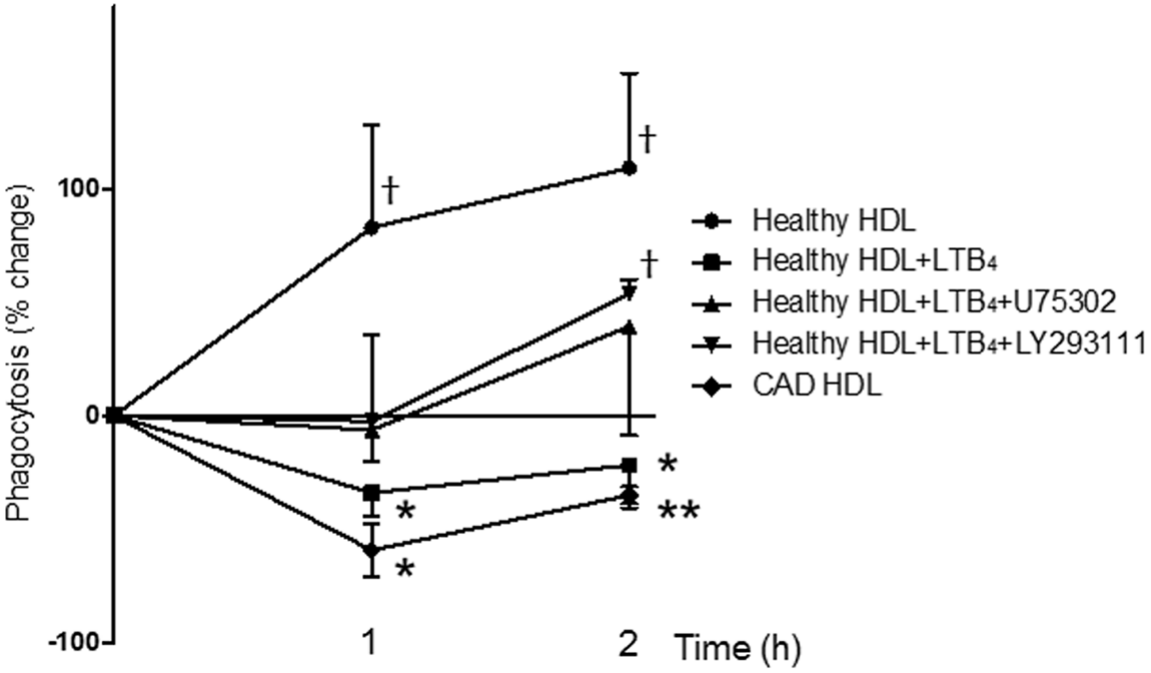
HDL_Healthy_ enhanced macrophage phagocytosis. Macrophages (0.5 × 10^5^) were pretreated with each HDL (10 µg) for 1 h or 2 h at 37 °C and then incubated with fluorescent-labelled zymosan for 30 min at 37 °C. In specific experiments, LTB4 (100 nM) or BLT1 antagonist LY293111 (300 nM) or U75302 (300 nM) were used as pretreatment. The results are percentage increases of phagocytosis compared with that after vehicle treatment and are shown as the mean ± SEM, N = 4. ^†^ *P* < 0.05 compared to vehicle. *P < 0.05, **P < 0.01, versus HDL_Healthy_.

After showing that HDL_CAD_ locally produced LTB_4_ *de novo* and that local LTB_4_ suppressed HDL engulfment into macrophages in a dose-dependent manner, we investigated the effect of the LTB_4_ receptor antagonist on HDL_CAD_-macrophage interactions. Macrophages were pretreated with the BLT1 antagonist U75302 (300 nM), or left untreated, before incubation with DiI-labelled HDL_Healthy_ and HDL_CAD_. Pretreatment with U75302 had no effect on HDL_Healthy_ engulfment into macrophages (Fig. 5a, upper panels and Fig. 6b, 1st-2nd lanes); however, HDL_CAD_ engulfment was significantly increased by U75302 pretreatment, and the particle engulfment was similar to that of HDL_Healthy_ (Fig. 5a, lower panels and Fig. 6b, 3rd-4th lanes).

### HDL_Healthy_ enhances macrophage phagocytosis

Because macrophage phagocytosis plays critical roles in the anti-atherogenic machinery^34,35^, we evaluated whether each HDL type might have distinct effects on macrophage phagocytosis. Pretreatment with HDL_Healthy_ significantly enhanced macrophage phagocytosis, as compared with treatment with the vehicle (Fig. 6). Notably, pretreatment with HDL_CAD_ and pretreatment with HDL_Healthy_ + LTB_4_ (100 nM) resulted in significant decreases in phagocytosis, and treatment with either BLT1 antagonist LY293111 or U75302 rescued macrophage phagocytosis. These results indicated that functional interactions between HDL_Healthy_ and macrophages contribute to enhanced macrophage efferocytosis, and local low-dose LTB_4_ may suppress these anti-atherogenic functions.

### Proposed HDL functions: regulation of LTB_4_ from activated macrophages via HDL holoparticle endocytosis

**Figure 7:**
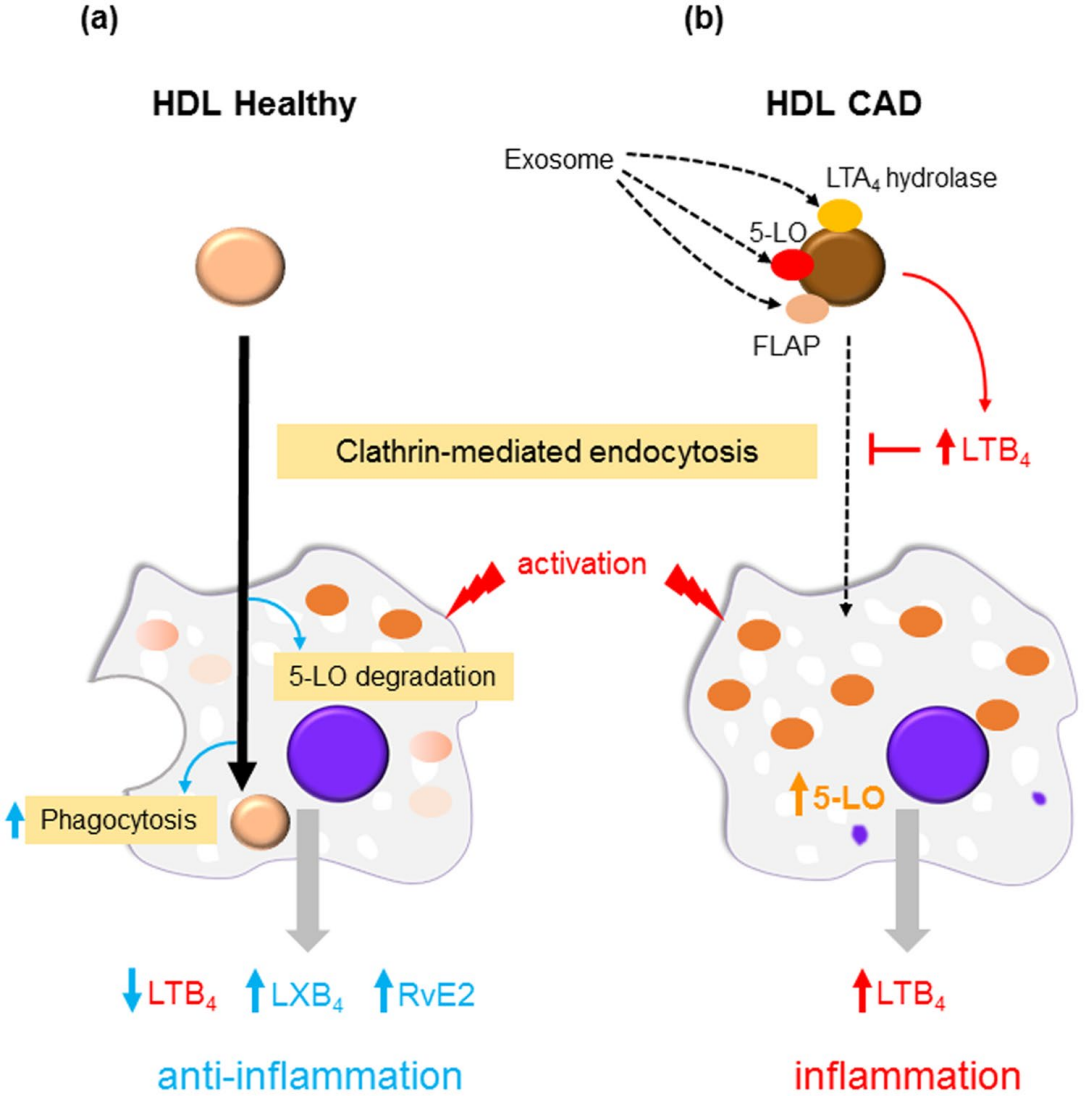
Proposed HDL functions: regulation of LTB_4_ production from activated macrophages via HDL engulfment. (**a**) HDL_Healthy_ is engulfed by macrophages via clathrin-mediated endocytosis and attenuates 5-LO expression by the ubiquitin proteasome system, thus resulting in decreased LTB_4_ from activated macrophages. HDL_Healthy_ enhances LXB_4_ and RvE2 production, as well as phagocytosis in macrophages. (**b**) HDL_CAD_ produces *de novo* LTB_4_, which interferes with HDL engulfment into macrophages. Here, 5-LO escapes from HDL-mediated degradation, thereby resulting in continuous LTB_4_ production from activated macrophages.

To address the functional interactions between HDL and macrophages, we propose novel HDL-initiated regulation of macrophages via HDL holoparticle endocytosis. HDL_Healthy_ was engulfed by macrophages via clathrin-mediated endocytosis and 5-LO expression attenuated by ubiquitin proteasome degradation, thus resulting in lower LTB_4_ production from activated macrophages (Fig. 7a). HDL_Healthy_ also enhanced LXB_4_ and RvE2 production, as well as phagocytosis in macrophages. In contrast, HDL_CAD_ carries neutrophil-like enzymatic machinery, which produced higher levels of local *de novo* LTB_4_. This enzymatic machinery may be transferred to HDL particle from neutrophil-derived exosomes. Locally produced *de novo* LTB_4_ interfered with HDL engulfment into macrophages (Fig. 7b). Here, 5-LO escaped from HDL-mediated degradation, thus resulting in continuous LTB_4_ production from activated macrophages.

## Discussion

In the present study, we demonstrated that HDL_Healthy_ contributes to anti-inflammatory and pro-resolving functions during HDL-macrophage interactions through HDL holoparticle endocytosis. We also found that HDL_CAD_ releases *de novo* pro-inflammatory LM LTB_4_, which locally interferes with the anti-inflammatory function of HDL by suppressing HDL particle engulfment into macrophages.

Only hepatocytes and endothelial cells have previously been reported to engulf HDL holoparticles^24–26^. Here, we reported human HDL engulfment into macrophages by tracking DiI-stained HDL (Fig. 2a) and human apoA-I protein expression within murine macrophages (Fig. 2b). After the addition of the clathrin inhibitor Pitstop 2, HDL engulfment was nearly completely abrogated (Fig. 2c), thus indicating that HDL engulfment is driven by clathrin mediated endocytosis. We observed that excess non-labelled HDL or apoA-I competitively decreased DiI-labelled HDL engulfment (Fig. 2e,f), thus suggesting the presence of an HDL-specific, apoA-I-dependent receptor for HDL engulfment. We next investigated the intracellular localization of HDL particles after engulfment into macrophages; however, we did not observe co-localization of HDL with early endosomes at 10-120 min after co-incubation (Supplemental Fig. 5a). HDL showed partial co-localization with lysosomes after 120 min co-incubation (Supplemental Fig. 5b). Further studies are required to identify the receptor for HDL engulfment and HDL trafficking within macrophages.

HDL engulfment into macrophages attenuated 5-LO expression by proteasome degradation (Fig. 1c,d), thus decreasing LTB_4_ production from activated macrophages (Fig. 1a,b). It remains unclear how HDL engulfment regulates proteasome-mediated degradation of 5-LO. Notably, engulfment of HDL_Healthy_ promoted anti-inflammatory, pro-resolving LM LXB_4_ and RvE2 release from macrophages (Table 1), and this release orchestrates resolution programs^20,21^, including enhanced phagocytosis of macrophages, as shown in Fig. 6. HDL_CAD_ did not enhance macrophage phagocytosis but instead resulted in decreased phagocytic functions. Recent studies have indicated that advanced atherosclerotic lesions are characterized by the pathological accumulation of diseased vascular cells and apoptotic cellular debris and that the removal of these cells and cellular debris appears to be significantly impaired in diseased blood vessels^34,35^. HDL_CAD_ may contribute to impaired macrophage phagocytosis and pathogenesis of atherosclerosis.

HDL carries several enzymes involved in lipid metabolism, such as lecithin-cholesterol acyltransferase and lipoprotein-associated phospholipase A_2_ (also known as platelet-activating factor acetylhydrolase)^36^. Using our LC/MS/MS-based metabololipidomics approach, we confirmed *de novo* LM production from HDL particles. Notably, we identified pro-inflammatory LTB_4_ production from HDL_CAD_ (Fig. 3a), as well as critical enzymes for LTB_4_ biosynthesis, particularly 5-LO, LTA_4_ hydrolase, and FLAP (Fig. 3c and Supplemental Fig. 6), which are enriched in activated neutrophils^37^. Our results indicated that the HDL_CAD_ carries LTB_4_-related enzymes and that this enzymatic machinery may be transferred to HDL_CAD_ via activated leukocyte-derived exosomes and microparticles, which may contain 5-LO, LTA_4_ hydrolase, and FLAP.

LTB_4_ promotes monocyte chemotaxis and conversion of monocytes to foam cells, thus resulting in accelerated atherosclerosis^38^. The LTB_4_ receptor (BLT1) is expressed in macrophages^39,40^. The contribution of the LTB_4_/BLT1 signalling pathway to atherosclerosis has been demonstrated by using a BLT1 antagonist and BLT1-deficient mice^41,42^. Additionally, 5-LO gene expression in peripheral blood mononuclear cells and LTB_4_ concentration in the plasma were augmented in patients with carotid atherosclerosis^43^. In our study, local *de novo* LTB_4_ release from HDL_CAD_ suppressed HDL engulfment into macrophages, thereby inhibiting HDL-mediated 5-LO degradation. The LTB_4_ antagonist U75302 rescued HDL engulfment (Figs 4a,b and 5a,b) and HDL-mediated 5-LO degradation (Fig. 4c) in macrophages, thus indicating a specific contribution of LTB_4_/BLT1 signalling pathways to HDL-macrophage functional interactions.

In summary, our results suggested that HDL holoparticle endocytosis plays critical roles in functional HDL-cell interactions. Additionally, HDL carries specific LM-producing enzymes that may be transferred from specific exosomes, and it releases *de novo* local LM, which controls HDL holoparticle engulfment and HDL-mediated regulation of cellular functions. Notably, *de novo* local LTB_4_ release from HDL_CAD_ interferes with HDL-macrophage interactions. The new pathway elucidated here suggests that therapeutic administration of an LTB_4_/BLT1 pathway antagonist may be beneficial for improving HDL-mediated anti-inflammatory and pro-resolving functions in cardiovascular disease patients.

## Methods

### Clinical participants and HDL preparation

HDL_Healthy_ (*n* = 4) and HDL_CAD_ (*n* = 4) were prepared from our previous study^14^, on the basis of serum MPO/PON1 ratios. Plasma was stored at −80 °C until use, HDL was isolated by ultracentrifugation as previously described^44^, and the purity of HDL isolation was confirmed by SDS-PAGE and subsequent Coomassie staining. This study was conducted in accordance with the Declaration of Helsinki. The study protocols complied with the Guidelines of the Ethical Committee of the Kobe University Graduate School of Medicine and was approved by the Institutional Review Board of Kobe University Graduate School of Medicine. Written informed consent for participation was obtained from all subjects before the study.

### LC/MS/MS-based LM metabololipidomics

Deuterated internal standards d_4_-LTB_4_, d_8_-5-HETE, d_4_-PGE_2_, and d_5_-RvD2, representing each chromatographic region of identified LMs, were added to the samples (500 pg each) to facilitate quantification. The samples were extracted by SPE on C18 columns as previously described^45^ and were subjected to LC-MS/MS. The system consisted of a Q-Trap 6500 (Sciex) equipped with a Shimadzu LC-30AD HPLC system. A ZORBAX Eclipse Plus C18 column (100 mm × 4.6 mm, 3.5 µm, Agilent Technologies) was used with a methanol/water/acetic acid gradient of 55:45:0.01 to 98:2:0.01 (v/v/v) at a 0.4 ml/min flow rate. For monitoring and quantifying the levels of targeted LMs, the multiple reaction monitoring (MRM) method was developed with signature ion pairs Q1 (parent ion)/Q3 (characteristic fragment ion) for each molecule. Identification was conducted with published criteria using the LC retention time, specific fragmentation patterns, and at least six diagnostic fragmentation ions. Quantification was carried out on the basis of the peak area of the MRM chromatograph, and the linear calibration curves were obtained with authentic standards for each compound.

### Macrophage-HDL interactions

RAW 264.7 macrophages were cultured in DMEM supplemented with 10% FBS. In total, 1 × 10^6^ macrophages were activated by opsonized ZyM particles (100 µg, 30 min at 37 °C) in PBS, and this was followed by co-incubation with HDL_Healthy_ or HDL_CAD_ (10 μg protein, 30 min at 37 °C). After incubation, a 2 × volume of ice-cold methanol was added for targeted LM metabololipidomics, and 200 µl of lysis buffer (20 mM HEPES (pH 7.4), 150 mM NaCl, 1% NP40, 1% SDS) was added for western blot analysis. The expression levels of 5-LO were monitored using an anti-5-LO antibody (3289, Cell Signaling Technology). In select experiments, macrophages were pretreated with the proteasome inhibitor MG132 (10012628, Cayman Chemical) before co-incubation with HDL.

### HDL engulfment into macrophages

Each HDL was stained with Dil (1704526, Molecular Probes) as previously described^46^. Briefly, HDL was incubated with CM-DiI for 15 min at 37 °C, and the mixture was dialyzed overnight to remove the residual staining solution. Macrophages (1 × 10^6^) were incubated with each DiI-labelled HDL (10 µg) for 30 min at 37 °C. HDL particle uptake into macrophages was investigated using confocal microscopy (LSM700, LEICA). For detection of human HDL-derived apoA-I from murine macrophages, macrophages (1 × 10^6^) were co-incubated with human apoA-I (SLBN8688V, SIGMA-ALDRICH, 10 µg) or each HDL (10 µg) for 30 min at 37 °C. After extensive washing with PBS, macrophages were harvested. Cell lysates were processed for western blot analysis of human apoA-I with an anti-human apoA-I antibody (23030485, CHEMICON INTERNATIONAL). In specific experiments, macrophages were pretreated with the clathrin-mediated endocytosis inhibitor Pitstop 2 (20 µM, ab120687, Abcam) for 30 min or the LTB_4_ receptor antagonist U75302 (300 nM, 70705, Cayman CHEMICAL). For analysis of the effects of LTB_4_ on HDL uptake into macrophages, the latter was pretreated with 1, 10, or 100 nM LTB_4_ (20110, Cayman CHEMICAL) for 30 min at 37 °C, and this was followed by incubation with DiI-labelled HDL_Healthy_ for 30 min. HDL uptake was analysed with confocal microscopy. In the same experiment, 5-LO protein expression in raw macrophages was also monitored by western blot analysis. After 2-h co-incubation with macrophages and HDL at 37 °C, the cells were washed in PBS, fixed with 4% formaldehyde for 15 min at 4 °C, and rinsed three times in PBS. The cells were permeabilized in 0.1% Triton X-100 for 10 min, blocked in 5% BSA/PBS for 1 h, and incubated with primary antibodies to apoA-I (23030485, CHEMICON INTERNATIONAL), EEA-1 (ab2900, Abcam) and LAMP-1 (816001, BioLegend) in 5% BSA/PBS overnight. Proteins were detected with Alexa Flour-labelled secondary antibodies.

### HDL-derived LM production

For analysis of HDL-derived LM production, each HDL (10 μg) was dialyzed with PBS^+/+^ to remove EDTA and then was incubated with 1 µM deuterium-labelled substrates (d-AA, d-EPA and d-DHA) for 2 h at 37 °C. Deuterium-labelled LMs were analysed with our targeted LM metabololipidomics. HDL-carrying proteins were analysed by western blotting. Briefly, 10 µg of each HDL was boiled for 5 min at 95 °C in SDS buffer, then subjected to SDS-PAGE. Specific proteins were detected using primary antibodies against 5-LO (3289, Cell Signaling) and LTA_4_ hydrolase (ab133512, Abcam).

### Detection of ubiquitinated 5-LO

ZyA-activated macrophages were pretreated for 30 min with MG 132 (1 µM) or vehicle, and this was followed by incubation with HDL_Healthy_ for 30 min at 37 °C. Lysates were collected with 1% SDS containing lysis buffer, boiled for 5 min and sonicated. Immunoprecipitation (IP) was performed with anti-5-LO Ab (#3289, Cell Signaling) using Dynabeads Protein A IP Kit (Thermo Fisher Scientific). After IP, western blot analysis was carried out with poly-UB antibody (ab140601, linkage-specific K48 antibody, Abcam).

### PCR analysis

Macrophage total RNA was extracted with TRIzol reagent (Invitrogen). cDNA was prepared from 1 µg total RNA by using PrimeScript RT reagent (RR047, Takara). Real-time polymerase chain reaction (real-time PCR) was performed with SYBR^TM^ Premix Ex Taq II (RR820, Takara). Primers were obtained from Takara Bio Inc. Amplification reactions were performed in duplicate using a LightCycler 96 Real-Time PCR system (Roche), and fluorescence curves were analysed with the included software. GAPDH was used as an internal control. Relative quantification was performed on the basis of the ΔΔCt method.

### Macrophage phagocytosis

Macrophages (0.5 × 10^5^) were cultured on 96-well plates and preincubated with or without 5 µg of HDL for 1 h or 2 h at 37 °C, and cells were treated with 5 µl of fluorescent-labelled opsonized zymosan (Molecular Probes Z2850) at a 10:1 ratio (zymosan:macrophages) for 30 min at 37 °C^47^. In specific experiments, LTB4 (100 nM) or BLT1 antagonist LY293111 or U75302 (300 nM) were used as pretreatment. After the incubations, macrophages were gently washed, extracellular fluorescence was quenched by addition of a 5-fold diluted trypan blue solution, and phagocytosis was measured with a fluorescent plate reader (EnSpire, PerkinElmer).

### Statistical Analysis

Results are expressed as the mean ± SEM. Statistical significance was determined using two-tailed Student’s *t* test for two-group comparisons and one-way ANOVA for multiple comparisons with post hoc analysis using Tukey’s test (GraphPad Prism). A *P* value < 0.05 was considered to be significant.

## Electronic supplementary material

Supplemental data
