# Supplementary material for: Novel mechanism of regulation of the 5-lipoxygenase/leukotriene B4 pathway by high-density lipoprotein in macrophages
Source: Sci Rep. 2017 Oct 11;7:12989. doi: 10.1038/s41598-017-13154-0 (PMC5636875; doi:10.1038/s41598-017-13154-0)

Supplementary Information

Novel mechanism of regulation of the 5-lipoxygenase/leukotriene B_4 pathway by high-density lipoprotein in macrophages

Shigeyasu Tsuda, Masakazu Shinohara, Toshihiko Oshita, Manabu Nagao, Nobuaki Tanaka, Takeshige Mori, Tetsuya Hara, Yasuhiro Irino, Ryuji Toh, Tatsuro Ishida, and Ken-ichi Hirata

Supplemental Method:

Preparation of mice bone marrow derived macrophages (BMDM).

Bone marrow cells were sterilely obtained from femoral bones of C57BL6/J mice and differentiated to macrophages in 10% FBS containing RPMI with supplementation of M-CSF (10 ng/mL) for seven days.

PCR analysis.

Macrophage total RNA was extracted with TRIzol reagent (Invitrogen). cDNA was prepared from 1 μg total RNA by using PrimeScript RT reagent (RR047, Takara). To confirm the expression of BLT1 in RAW macrophages, PCR amplification for mouse BLT1 was performed with and without reverse transcription (RT). Primers for mouse BLT1 were Forward: CCTGGCACTAAGACAGATTCAAGGA, Reverse: CCTGGCACTAAGACAGATTCAAGGA, product size was 152bp. PCR product was separated by electrophoresis with 2% agarose gel.

Isolation of exosomes from plasma.

Exosomes were isolated from plasma with ExoTrap™ (SHI-EXO-K010, COSMO BIO). Exosome-derived proteins were eluted by SDS sample buffer, denatured by heating and applied for western blot analysis with anti-CD9 antibody (12A12, COSMO BIO) and anti-ApoA-I antibody (23030485, CHMEICON INTERNATIONAL)

Supplemental Tables:

Supplementary Table 1. Subject characteristics

	Healthy group	CAD group
Sex	4M	3M/1F
Age (years)	32 - 42	52 - 69
Serum MPO mass (ng/ml)	7.2 ± 6.4	275.7 ± 17.8*
Serum PON1 activity (U/L)	253.0 ± 40.1	120.7 ± 62.7*
Serum MPO/PON1	0.03 ± 0.02	2.89 ± 1.78*
EPA/AA in HDL	0.42 ± 0.02	0.25 ± 0.10
DHA/AA in HDL	0.58 ± 0.02	0.56 ± 0.19
DPA/AA in HDL	0.10 ± 0.02	0.12 ± 0.02

Values were represented as mean ± SEM. *$P < 0.05$ between two groups.

Supplementary Table 2. Lipid mediator biosynthesized from $HDL_{Healthy}$ or HDL_{CAD}

	$HDL_{Healthy}$ (pg/0.01mg protein/2h)	HDL_{CAD} (pg/0.01mg protein/2h)
AA Bioactive Metabolome		
Lipoxin A_4	-	-
Lipoxin B_4	-	-
Prostaglandin D_2	3.5 ± 1.1	23.5 ± 8.6*
Prostaglandin E_2	2.0 ± 0.2	4.4 ± 1.9
Prostaglandin $F_{2α}$	-	-
Thromboxane B_2	-	-
Leukotriene B_4	0.4 ± 0.1	20.0 ± 21.2*
AA pathway markers		
5 HETE	0.7 ± 0.2	7.2 ± 8.8*
12 HETE	5.9 ± 1.2	52.9 ± 33.0
15 HETE	4.1 ± 0.7	7.8 ± 7.2
EPA pathway markers		
5 HEPE	2.6 ± 0.7	11.3 ± 0.9*
12 HEPE	20.5 ± 2.2	11.8 ± 4.9
15 HEPE	2.3 ± 0.3	5.5 ± 3.6
18 HEPE	2.3 ± 0.2	10.2 ± 4.6
DHA pathway markers		
4 HDHA	1.3 ± 0.4	5.5 ± 2.0*
7 HDHA	0.7 ± 0.1	2.8 ± 0.6*
14 HDHA	6.7 ± 1.1	8.0 ± 1.9
17 HDHA	1.3 ± 0.7	5.4 ± 2.1

- = below detection limits.
Values are represented as mean ± SEM. *P < 0.05 between two groups.

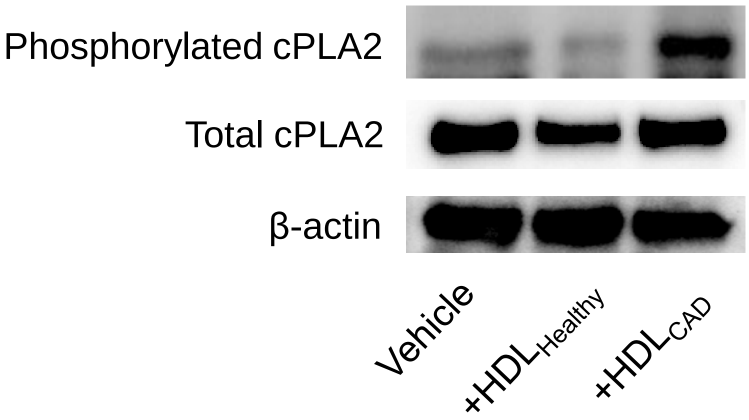

Supplemental Figure 1. HDL_{CAD} enhanced phosphorylation of cPLA2 in macrophages.

Macrophages (1×10^6) were incubated with vehicle or HDL (Healthy or CAD, 10 µg protein, 30 min at 37 ºC). After incubation, 200 µl of lysis buffer (20 mM HEPES (pH 7.4), 150 mM NaCl, 1% NP40, 1% SDS) was added for western blot analysis. The expression levels of phosphorylation of cPLA2 and total cPLA2 were analyzed using an anti-phospho-cPLA2 antibody (2831, Cell Signaling Technology) and cPLA2 (5249, Cell Signaling Technology) .

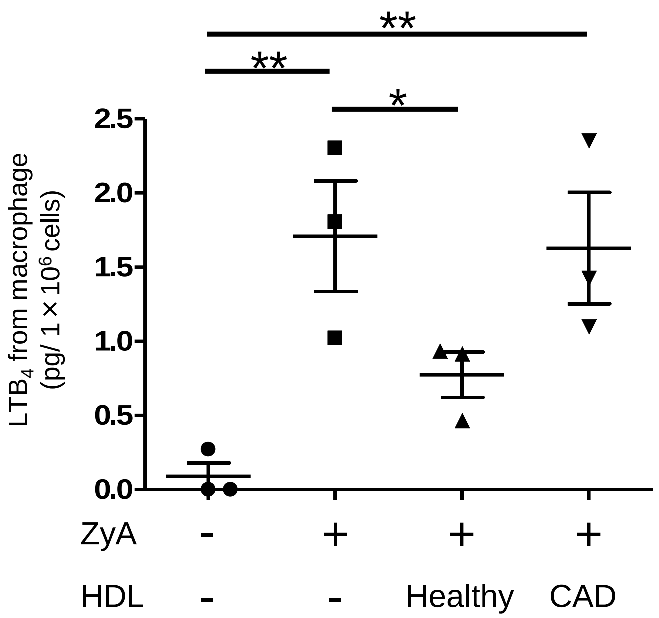

Supplemental Figure 2. $HDL_{Healthy}$, but not HDL_{CAD}, decreased LTB_4 production from mouse bone marrow-derived macrophages.

Mouse bone marrow-derived macrophages (1×10^6) were activated by ZyA (30 min at 37 ºC), then treated with $HDL_{Healthy}$ or HDL_{CAD} (10 μg protein). LTB_4 production from macrophages was quantified by LC/MS/MS. The results are expressed as pg/1×10^6 cells, mean ± SEM, N = 3 in each group.

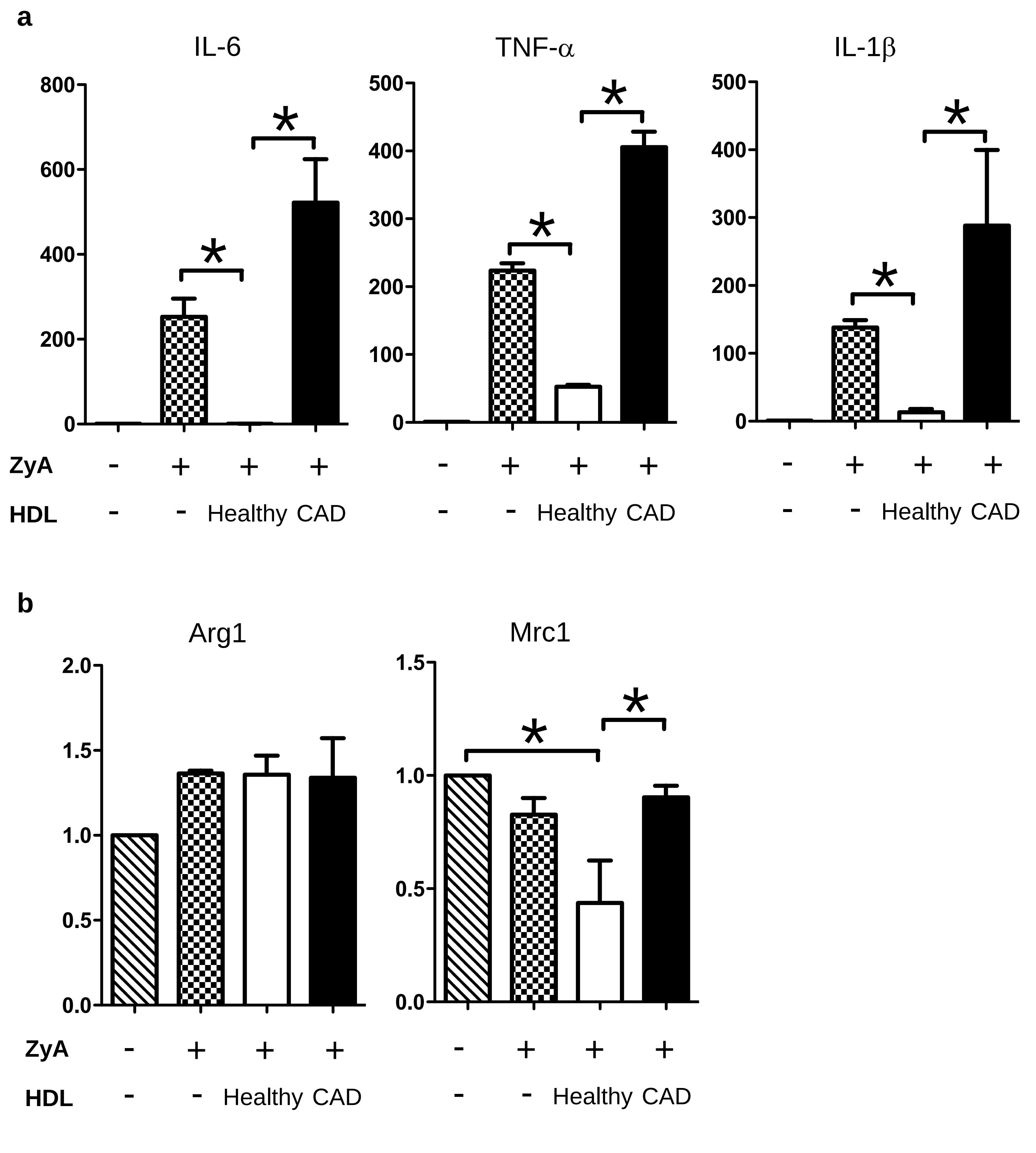

Supplemental Figure 3. Macrophages showed proinflammatory cytokine profiles by HDL_{CAD}.

Macrophages (RAW 264.7 cell line, $1{\times}10^6$) were activated by ZyA (30 min at 37ºC), then treated with $HDL_{Healthy}$ or HDL_{CAD} (10 μg protein, 30 min at 37ºC). After incubation, total RNA was extracted, then M1 markers (**a**: IL-6, TNF-α, IL-1β) and M2 markers (**b**: Arg1, Mrc1) were analyzed by real-time PCR. The results are shown as fold change compared to vehicle group; mean ± SEM, N = 3. *$P < 0.05$ by post hoc analysis.

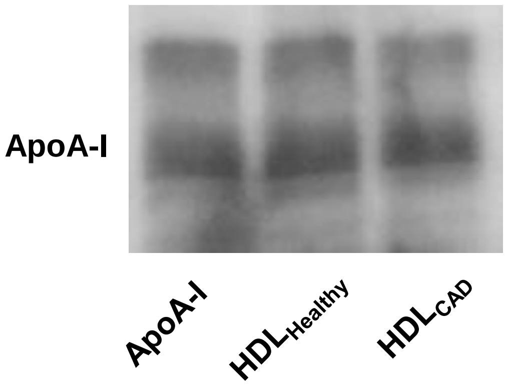

Supplemental Figure 4. ApoA-I expression levels in $HDL_{Healthy}$ and HDL_{CAD}.

Each type of HDL was purified by ultracentrifuge. Apo-AI (SLBN8688V, SIGMA-ALDRICH), $HDL_{Healthy}$,and HDL_{CAD} (10µg protein, respectively) were investigated by western blot analysis with anti-ApoA-I (#23030485, CHEMICON INTERNATIONAL).

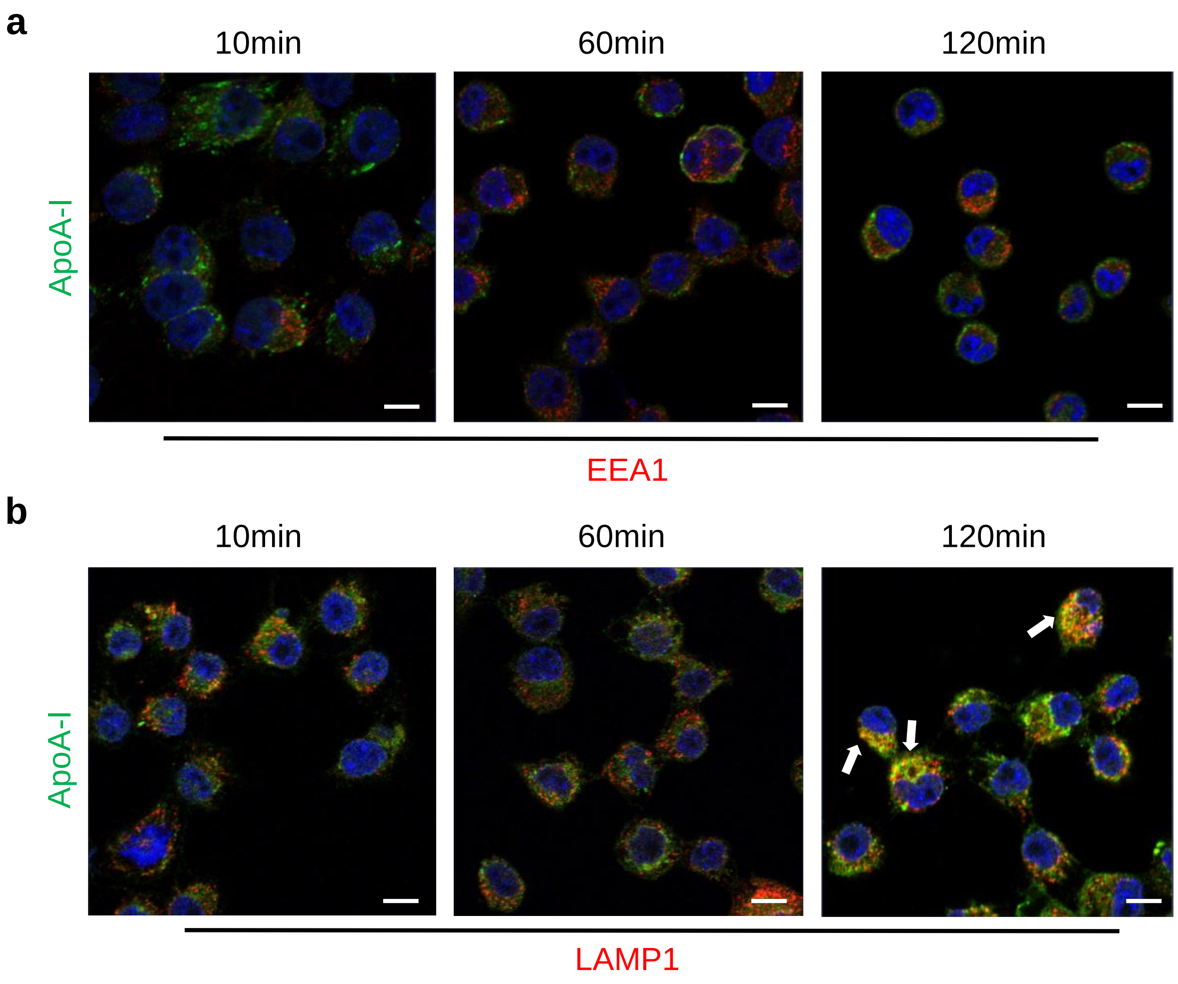

Supplemental Figure 5. HDL partially colocalized with lysosomes in macrophages.

Macrophages were incubated with 10 µg of $HDL_{Healthy}$ at 37ºC for 10, 60 and 120 min. The cells were fixed and immunostained with (**a**) anti-EEA1 (shown as red) and anti-human apoA-I (shown as green) antibodies or (**b**) anti-LAMP1 (shown as red) and anti-human apoA-I (shown as green) antibodies, followed by DAPI staining (shown as blue). Scale bars = 10 µm. The arrows indicate apoA-I colocalization with lysosomes.

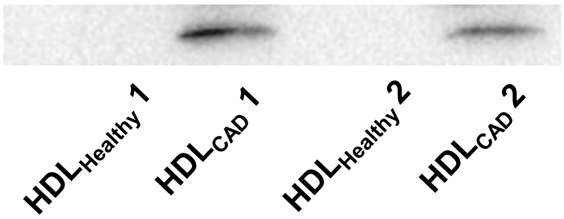

Supplemental Figure 6. HDL_{CAD} contained FLAP.

Each type of HDL was purified by ultracentrifuge. $HDL_{Healthy}$ and HDL_{CAD} (10 µg protein, respectively) were investigated by western blot analysis with anti-FLAP antibody (ab85227, abcam).

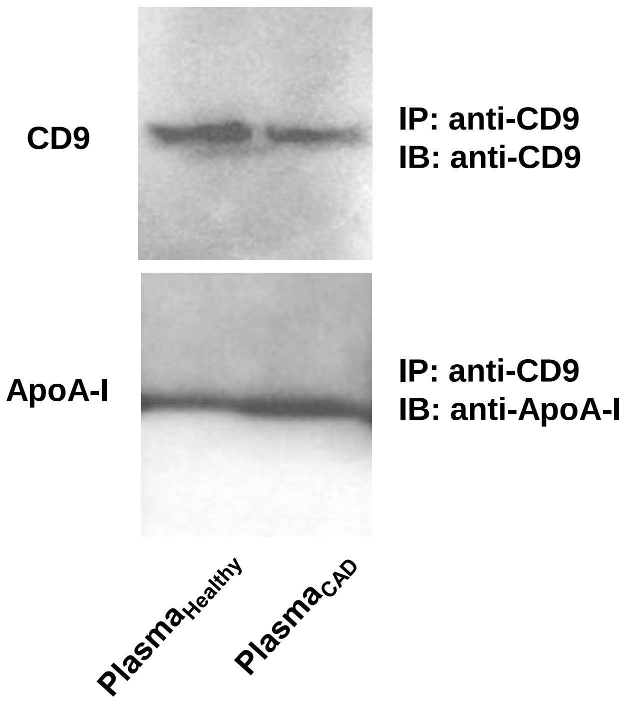

Supplemental Figure 7. HDL contained exosomes in circulating plasma.

$Plasma_{Healthy}$ and $Plasma_{CAD}$ were immunoprecipitated with anti-CD9 antibody (12A12, Cosmo BIo) using ExoTrap^{TM} Exosome Isolation Spin Column Kit (Cosmo Bio), then immunoblotted with anti-CD9 antibody antibody (12A12, Cosmo Bio) and anti-ApoA-I antibody (#23030485, CHEMICON INTERNATIONAL).

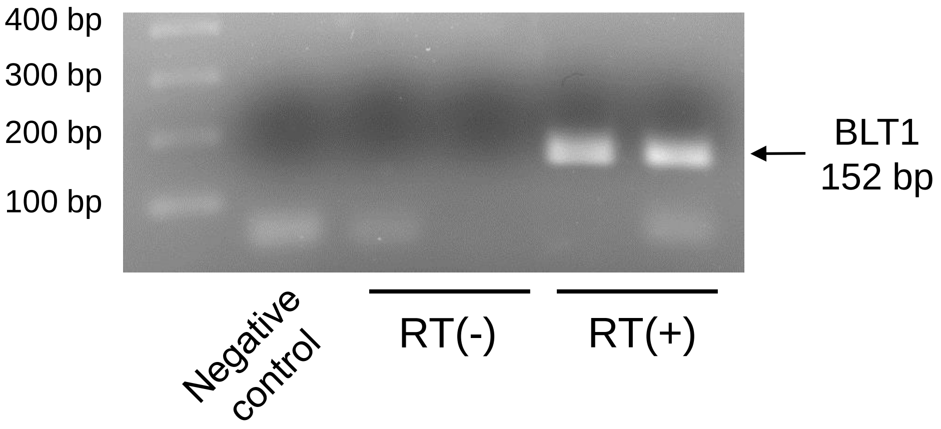

Supplemental Figure 8. RAW macrophages expressed BLT1.

Total RNA was extracted from zymosan stimulated RAW macrophages. PCR amplification for mice BLT1 was performed with or without reverse transcription (RT). PCR products were separated by electrophoresis with 2% agarose gel.

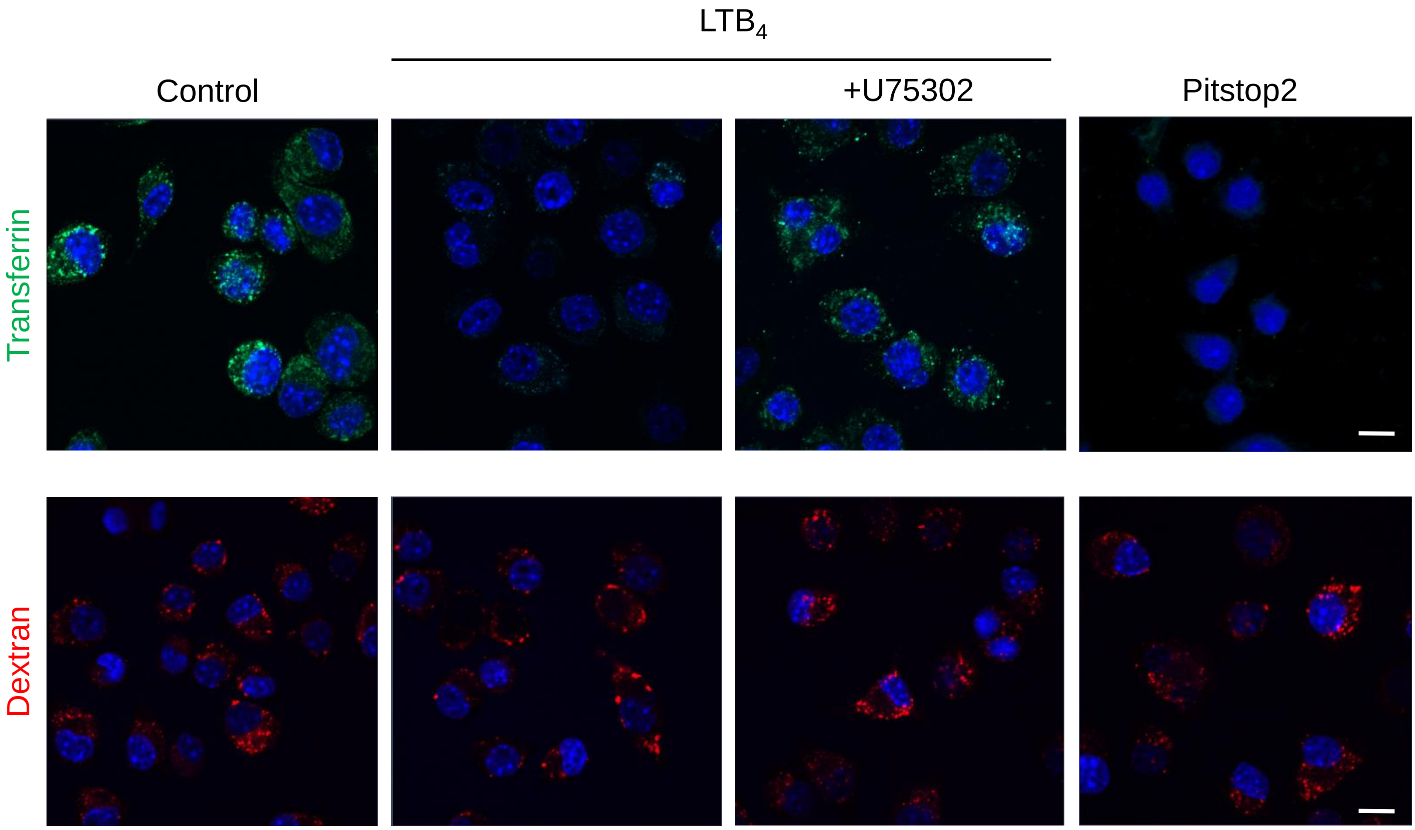

Supplemental Figure 9. LTB_4 selectively attenuated clathrin-mediated endocytosis.

Macrophages were pretreated for 30 min at 37ºC with vehicle, LTB_4 (100 nM) with/without BLT1 antagonist U75302 (300 nM), or the clathrin-mediated endocytosis inhibitor Pitstop2 (20 μM). Next, macrophages were co-incubated with 10 μg/ml transferrin (shown as *green*) or dextran (shown as *red*) for 30 min at 37 ºC, followed by DAPI staining (shown as blue) (scale bars = 10 μm).

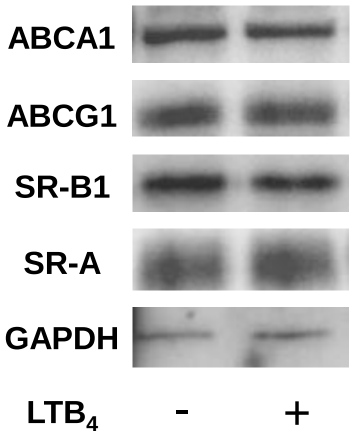

Supplemental Figure 10. LTB$_4$ treatment did not affect expression of ApoA-I receptors.

Macrophages were treated with or without 100 nM LTB$_4$ for 2hr at 37ºC. Cell lysates were processed for western blot analysis with anti-ABCA1 antibody (NB400-105, Novus Biologicals), anti-ABCG1 antibody (NB400-132 , Novus Biologicals), anti-SR-B1 antibody (NB400-104 , Novus Biologicals), and anti-SR-A antibody (CD204, Trans Genic).

Supplemental Figure 11. Gels presented in the main figures.

Figure 1c

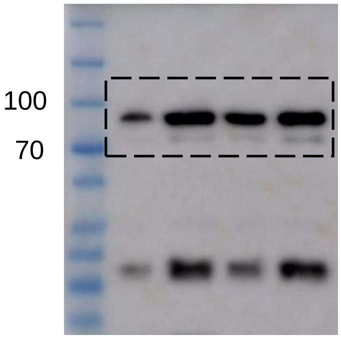

Figure 1c
β-actin

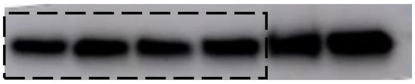

Figure 1d

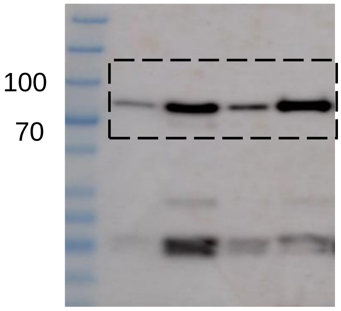

Figure 1d
β-actin

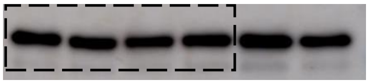

Figure 2b

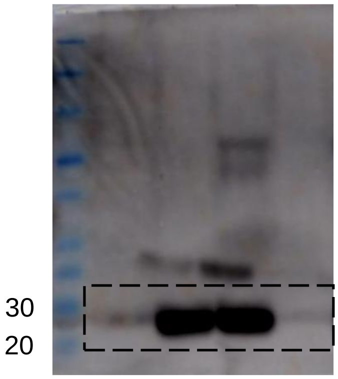

Figure 2d (ApoA-I)

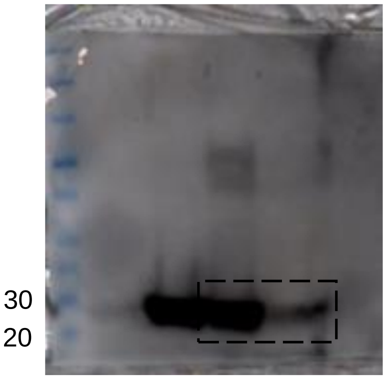

Figure 2d (5-LO)

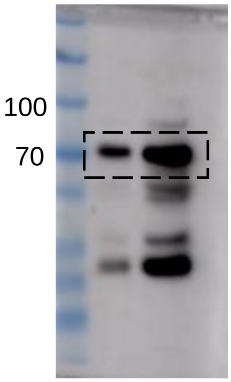

Figure 3c (5-LO)

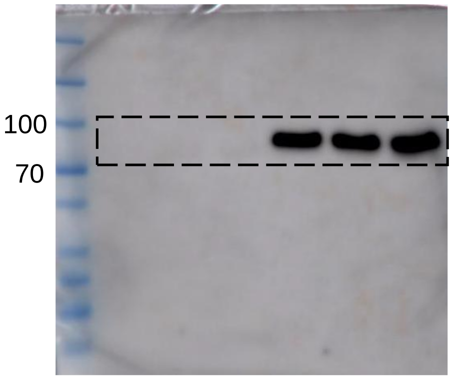

Figure 3c
(LTA4 hydrolase)

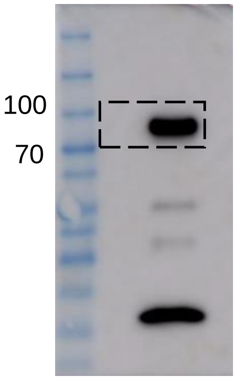

Figure 4c (5-LO)

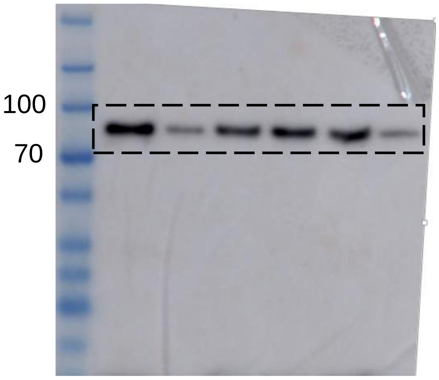

Figure 4c (β-actin)

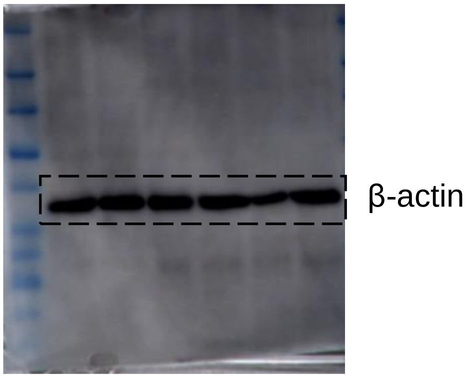

Supplement: Supplementary file 1 — Supplemental data [file 41598_2017_13154_MOESM1_ESM.pdf]
